# Prevalence of Immunosuppressive Drug Use Among Commercially Insured US Adults, 2018-2019

**DOI:** 10.1001/jamanetworkopen.2021.4920

**Published:** 2021-05-20

**Authors:** Beth I. Wallace, Brooke Kenney, Preeti N. Malani, Daniel J. Clauw, Brahmajee K. Nallamothu, Akbar K. Waljee

**Affiliations:** 1 Department of Internal Medicine, University of Michigan Medical School, Ann Arbor; 2 VA Center for Clinical Management Research, VA Ann Arbor Healthcare System, Ann Arbor, Michigan; 3 Department of Surgery, University of Michigan Medical School, Ann Arbor; 4 Department of Anesthesiology, University of Michigan Medical School, Ann Arbor

## Abstract

This cross-sectional study uses direct pharmaceutical claims data from a commercial claims database to assess the use of 6 types of immunosuppressive drugs among commercially insured US adults in 2018 and 2019.

## Introduction

The use of immunosuppressive drugs is a potential risk factor for infectious disease, including COVID-19 illness.^1^ For instance, although dexamethasone improves survival among patients with severe COVID-19 infections,^2^ long-term glucocorticoid use may increase the risk of hospitalization among patients who contract COVID-19.^3^ A prior study relying on patient self-report estimated that 2.7% of US adults were immunosuppressed in 2013, but the study did not explore features associated with use of immunosuppressive medications.^4^ In this cross-sectional study, we used direct pharmaceutical claims to describe the contemporary prevalence of drug-induced immunosuppression in a large cohort of US adults.

## Methods

Deidentified data from Clinformatics Data Mart (Optum, Inc), a national commercial claims database, were used in this cross-sectional study. Adults aged 18 through 64 years who had continuous commercial medical insurance coverage from January 1, 2017, through December 31, 2019, were included. We used 2017 data to establish baseline comorbid conditions; 2018 and 2019 data were used to determine drug-induced immunosuppression. We defined drug-induced immunosuppression as use of any of the following regimens in a 365-day period: (1) 1 dose or more of an antineoplastic immunosuppressive drug; (2) 30 days or more of oral glucocorticoids; (3) 90 days of any other oral or subcutaneous immunosuppressive drug; or (4) 2 doses of intravenous, noncorticosteroid immunosuppressive drugs. We excluded single-dose intravenous corticosteroids, which are often used as premedication for infusions. We classified immunosuppressive drugs into 6 categories as follows: oral corticosteroids, methotrexate, other disease-modifying antirheumatic drugs and transplant antirejection medications, tumor necrosis factor inhibitors, antineoplastic agents, and other biological product medications and Janus kinase inhibitors (eTable 1 in the Supplement). We used Agency for Healthcare Research and Quality Clinical Classifications Software Refined (CCSR) to group *International Statistical Classification of Diseases and Related Health Problems, Tenth Revision* diagnosis codes for common medical conditions associated with drug-induced immunosuppression and common primary diagnoses (eTable 2 in the Supplement). This study was granted exempt status, including a waiver of informed patient consent for use of deidentified secondary data, by the University of Michigan Institutional Review Board. This study followed the Strengthening the Reporting of Observational Studies in Epidemiology (STROBE) reporting guideline for cross-sectional studies.

## Results

Of the 3 169 441 continuously enrolled patients, 89 925 (2.8%) met the criteria for drug-induced immunosuppression during the period January 1, 2018, through December 31, 2019. Table 1 shows baseline patient characteristics. Most recipients of immunosuppressive drugs were older (median age, 53 years; interquartile range, 50-59 years), and women (55 043 [61.2%]).The most commonly prescribed immunosuppressive drugs were prednisone (47 649 patients [53.0%]), methotrexate (22 013 [24.5%]), and methylprednisolone (19 405 [21.6%]), which together were used by 62.5% of patients (Table 2). Oral corticosteroids were received by 67.7% of patients, and 40.9% received oral corticosteroids for 30 days or longer in a 365-day period. The 3 most common immunosuppression-associated diagnosis categories were malignant neoplasms (73.8%), immune-mediated conditions (68.8%), and inflammatory skin conditions (38.8%). After excluding 1 primary diagnosis category that was too general to provide useful diagnostic classification (FAC014, “medical examination/evaluation”), the 3 most common primary diagnosis categories were “neoplasm-related encounters” (51.8%), “exposure, encounters, screening, or contact with infectious disease” (50.6%), and “musculoskeletal findings, not low back pain” (48.3%).

**Table 1.** Baseline Characteristics of Patients Experiencing Drug-Induced Immunosuppression, 2018-2019

Characteristic	No. (%) (N = 89 925)
Age, y	
18-35	10 350 (11.5)
36-45	14 478 (16.1)
46-55	27 516 (30.6)
56-64	37 581 (41.8)
Men	34 882 (38.8)
Women	55 043 (61.2)
Elixhauser comorbidity index score, median (interquartile range)^a^	2 (1-4)
Highest level of education	
<High school	370 (0.4)
High school	22 643 (25.2)
<Bachelor’s degree	46 398 (51.6)
≥Bachelor’s degree	16 555 (18.4)
Unknown	3959 (4.4)
US Census region	
Midwest	22 137 (24.6)
Northeast	8177 (9.1)
South	41 808 (46.5)
West	17 635 (19.6)
Unreported	168 (0.2)

^a^ The Elixhauser comorbidity index assesses 30 comorbidity groups as present or absent, generating a numeric score that can be used for population risk adjustment. A score of 2 means 2 of the 30 assessed comorbidity groups are present.

**Table 2.** Most Commonly Prescribed Immunosuppressive Medications and Medical Diagnoses Among Patients Experiencing Drug-Induced Immunosuppression, 2018-2019

Medications and diagnoses	No. (%) (N = 89 925)^a^
Drug class	
1. Oral corticosteroids^b^	60 860 (67.7)
2. Methotrexate	22 013 (24.5)
3. Nonbiological DMARDs and transplant antirejection medications	23 315 (25.9)
4. TNF inhibitors	20 434 (22.7)
5. Other biological product medications^c^	12 276 (13.7)
6. Antineoplastic medications	16 191 (18.0)
Most common immunosuppression-associated diagnoses	
1. Malignant neoplasm	66 385 (73.8)
2. Immune-mediated conditions	61 822 (68.8)
3. Inflammatory skin conditions	34 895 (38.8)
4. Asthma or COPD	24 205 (26.9)
5. Organ transplant^d^	7105 (7.9)
Most common primary diagnoses	
1. Neoplasm-related encounters	46 624 (51.8)
2. Exposure, encounters, screening, or contact with infectious disease	45 489 (50.6)
3. Musculoskeletal pain, not low back pain	43 457 (48.3)
4. Respiratory signs and symptoms	33 019 (36.7)
5. Abdominal pain and other digestive/abdomen signs and symptoms	31 509 (35.0)

Abbreviations: COPD, chronic obstructive pulmonary disease; DMARD, disease-modifying antirheumatic drug; TNF, tumor necrosis factor.

^a^ The total number of prescriptions for the study period was 1 455 458.

^b^ This group of drugs includes oral formulations of prednisone, prednisolone, methylprednisolone, dexamethasone, cortisone, and hydrocortisone.

^c^ This class includes Janus kinase inhibitors.

^d^ This diagnosis group includes solid organ, skin, cornea, bone, bone marrow, and stem cell transplant.

## Discussion

In a national cohort of insured adults, more than 80 000 patients (2.8%) experienced drug-induced immunosuppression during the study period. Approximately two-thirds of patients (67.7%) received oral corticosteroids, and nearly half (40.9%) used oral corticosteroids for 30 days or longer. Common primary diagnoses in the study population included conditions often associated with corticosteroid use (eg, infections) and conditions for which corticosteroids are often prescribed despite limited evidence of benefit (eg, musculoskeletal pain, respiratory ailments).^5^ Limitations of this study include the uncertainty regarding the exact drugs prescribed for a given condition, dual indications for certain drugs (eg, methotrexate, cyclophosphamide), incomplete capture of inpatient medications, imperfect generalizability of commercial claims data, lack of corticosteroid dose information, and lack of modeling to demonstrate associations.

These findings are noteworthy given evolving evidence that long-term glucocorticoid use may increase the risk of COVID-19–related hospitalization.^3^ Steroid-sparing immunosuppressants may present an alternative to long-term corticosteroid use, but limited data exist regarding how these treatments affect the risk of hospitalization for COVID-19.^3^ While the possibility of an association is being clarified, clinicians should adhere to principles of corticosteroid stewardship by avoiding unnecessary corticosteroid prescribing when possible; carefully discussing the risks, benefits, and available treatment alternatives with patients; using the lowest drug dose and shortest duration appropriate for the condition; and advocating for public health measures.^6^

## Notes

Supplement. **eTable 1.** Categorization of Immunosuppressive Drugs Included in Search **eTable 2.** Diagnosis Categorizations by CCSR ICD-10 Groupings
