# Supplementary material for: Prevalence of Immunosuppressive Drug Use Among Commercially Insured US Adults, 2018-2019
Source: JAMA Netw Open. 2021 May 20;4(5):e214920. doi: 10.1001/jamanetworkopen.2021.4920 (PMC8138687; doi:10.1001/jamanetworkopen.2021.4920)
Supplement: Supplement. — eTable 1. Categorization of Immunosuppressive Drugs Included in Search eTable 2. Diagnosis Categorizations by CCSR ICD-10 Groupings [file jamanetwopen-e214920-s001.pdf]

# SUPPLEMENTARY ONLINE CONTENT

Wallace BI, Kenney B, Malani PN, Clauw DJ, Nallamothu BK, Waljee AK. Prevalence of immunosuppressive drug use among commercially insured US adults, 2018-2019. *JAMA Netw Open.* 2021;4(5):e214920. doi:10.1001/jamanetworkopen.2021.4920

**eTable 1.** Categorization of Immunosuppressive Drugs Included in Search

**eTable 2.** Diagnosis Categorizations by CCSR ICD-10 Groupings

This supplementary material has been provided by the authors to give readers additional information about their work.

© 2021 Wallace BI et al. *JAMA Network Open.*

**eTable 1.** Categorization of Immunosuppressive Drugs Included in Search

| | | |
|---|---|---|
| Oral steroids | BUDESONIDE | METHYLPREDNISOLONE |
| | DEXAMETHASONE | PREDNISONE |
| | HYDROCORTISONE | PREDNISOLONE |
| Methotrexate | | |
| Other nonbiologic DMARDs and transplant anti-rejection medications | APREMILAST | MERCAPTOPURINE |
| | AZATHIOPRINE | MYCOPHENOLATE MOFETIL |
| | CYCLOSPORINE | MYCOPHENOLIC ACID |
| | EVEROLIMUS | SIROLIMUS |
| | LEFLUNOMIDE | TACROLIMUS |
| TNF Inhibitors | ADALIMUMAB | INFLIXIMAB |
| | CERTOLIZUMAB | INFLIXIMAB ADBA |
| | ETANERCEPT | INFLIXIMAB DYYB |
| | GOLIMUMAB | |
| Other Biologic Medications and Janus Kinase Inhibitors | ABATACEPT | MUROMONAB-CD3 |
| | ANAKINRA | OMALIZUMAB |
| | BARICITINIB | UPADACITINIB |
| | BASILIXIMAB | RISANKIZUMAB |
| | BELATACEPT | RESLIZUMAB |
| | BELIMUMAB | RILONACEPT |
| | BENRALIZUMAB | SARILUMAB |
| | BEZLOTOXUMAB | SECUKINUMAB |
| | BRODALUMAB | SILTUXIMAB |
| | CANAKINUMAB | TILDRAKIZUMAB |
| | GUSELKUMAB | TOCILIZUMAB |
| | IXEKIZUMAB | TOFACITINIB |
| | MEPOLIZUMAB | USTEKINUMAB |
| Antineoplastic Medications | ABEMACICLIB | GLASDEGIB |
| | ABIRATERONE | IBRUTINIB |
| | ACALABRUTINIB | IDARUBICIN |
| | AFATINIB | IDELALISIB |
| | ALECTINIB | IFOSFAMIDE |
| | ALPELISIB | IXAZOMIB |
| | AXITINIB | IMATINIB MESYLATE |
| | AZACITIDINE | LAROTRECTINIB |
| | BEXAROTENE | LENALIDOMIDE |
| | BINIMETINIB | LORLATINIB |
| | BLEOMYCIN | MECHLORETHAMINE |

© 2021 Wallace BI et al. *JAMA Network Open.*

| | | |
|---|---|---|
| | BOSUTINIB | MELPHALAN HCL |
| | BRIGATINIB | MIDOSTAURIN |
| | BUSULFAN | MITOMYCIN |
| | CABOZANTINIB | NILOTINIB |
| | CAPECITABINE | NIRAPARIB |
| | CARMUSTINE | NIVOLUMAB |
| | CERITINIB | LENVATINIB |
| | CHLORAMBUCIL | LOMUSTINE |
| | CLADRIBINE | PANOBINOSTAT |
| | COBIMETINIB | PEMETREXED |
| | CRIZOTINIB | OLAPARIB |
| | CYCLOPHOSPHAMIDE | OMACETAXINE MEPESUCCINATE |
| | CYTARABINE | OSIMERTINIB |
| | DABRAFENIB | PALBOCICLIB |
| | DACTINOMYCIN | PAZOPANIB |
| | DACOMITINIB | PEXIDARTINIB |
| | DAROLUTAMIDE | POMALIDOMIDE |
| | DASATINIB | PONATINIB |
| | DAUNORUBICIN | PROCARBAZINE |
| | DAUNORUBICIN/CYTARABINE | REGORAFENIB |
| | DOXORUBICIN | RIBOCICLIB |
| | DUVELISIB | RUCAPARIB |
| | ENCORAFENIB | RUXOLITINIB |
| | ENTRECTINIB | SELINEXOR |
| | ERDAFITINIB | SORAFENIB |
| | ESTRAMUSTINE | STREPTOZOCIN |
| | ETOPOSIDE | SUNITINIB |
| | EXEMESTANE | TALAZOPARIB |
| | FEDRATINIB | THALIDOMIDE |
| | FLUDARABINE | THIOGUANINE |
| | FLUOROURACIL | THIOTEPA |
| | FLUTAMIDE | TIPIRACIL/TRIFLURIDINE |
| | GEFITINIB | VENETOCLAX |
| | GILTERITINIB | ZIV-AFLIBERCEPT INK |

© 2021 Wallace BI et al. *JAMA Network Open.*

**eTable 2.** Diagnosis Categorizations by CCSR ICD-10 Groupings

| Category | CCSR diagnostic groupings | |
|---|---|---|
| Immune-mediated conditions | Other specified connective tissue disease | Multiple sclerosis |
| | Rheumatoid arthritis and related disease | Juvenile arthritis |
| | Systemic lupus erythematosus and connective tissue disorders | Immune-mediated/reactive arthropathies |
| | Regional enteritis and ulcerative colitis | Autoinflammatory syndromes |
| | Other specified chronic arthropathy | Noninfectious hepatitis |
| | Uveitis and ocular inflammation | |
| Inflammatory skin conditions | Other specified inflammatory condition of skin | |
| Organ transplant | Organ transplant status | |
| Asthma and COPD | Asthma | Chronic obstructive pulmonary disease and bronchiectasis |
| Malignancy | Neoplasm-related encounters | Female reproductive system cancers - endometrium |
| | Neoplasms of unspecified nature or uncertain behavior | Female reproductive system cancers - cervix |
| | Encounter for antineoplastic therapies | Gastrointestinal cancers - esophagus |
| | Secondary malignancies | Head and neck cancers - lip and oral cavity |
| | Breast cancer - all other types | Gastrointestinal cancers - peritoneum |
| | Conditions due to neoplasm or the treatment of neoplasm | Male reproductive system cancers - testis |
| | Gastrointestinal cancers - colorectal | Female reproductive system cancers - uterus |
| | Malignant neoplasm, unspecified | Gastrointestinal cancers - bile duct |
| | Non-Hodgkin lymphoma | Nervous system cancers - all other types |
| | Respiratory cancers | Urinary system cancers - ureter and renal pelvis |
| | Breast cancer - ductal carcinoma in situ (DCIS) | Female reproductive system cancers - all other types |
| | Leukemia - all other types | Head and neck cancers - laryngeal |
| | Multiple myeloma | Female reproductive system cancers - vulva |
| | Male reproductive system cancers - prostate | Endocrine system cancers - all other types |
| | Leukemia - chronic myeloid leukemia (CML) | Head and neck cancers - throat |
| | Gastrointestinal cancers - liver | Female reproductive system cancers - fallopian tube |
| | Urinary system cancers - kidney | Head and neck cancers - eye |
| | Leukemia - chronic lymphocytic leukemia (CLL) | Head and neck cancers - tonsils |
| | Urinary system cancers - bladder | Head and neck cancers - pharyngeal |
| | Gastrointestinal cancers - anus | Urinary system cancers - all other types |

© 2021 Wallace BI et al. *JAMA Network Open.*

| | | |
|---|---|---|
| | Endocrine system cancers - thyroid | Head and neck cancers - salivary gland |
| | Nervous system cancers - brain | Leukemia - hairy cell |
| | Endocrine system cancers - pancreas | Head and neck cancers - nasopharyngeal |
| | Hodgkin lymphoma | Cardiac cancers |
| | Sarcoma | Endocrine system cancers - thymus |
| | Leukemia - acute myeloid leukemia (AML) | Female reproductive system cancers - vagina |
| | Myelodysplastic syndrome (MDS) | Gastrointestinal cancers - gallbladder |
| | Female reproductive system cancers - ovary | Endocrine system cancers - pituitary gland |
| | Cancer of other sites | Head and neck cancers - hypopharyngeal |
| | Gastrointestinal cancers - all other types | Mesothelioma |
| | Gastrointestinal cancers - stomach | Male reproductive system cancers - all other types |
| | Bone cancer | Urinary system cancers - urethra |
| | Malignant neuroendocrine tumors | Endocrine system cancers - adrenocortical |
| | Leukemia - acute lymphoblastic leukemia (ALL) | Male reproductive system cancers - penis |
| | Gastrointestinal cancers - small intestine | Endocrine system cancers - parathyroid |
| | Head and neck cancers - all other types | |

© 2021 Wallace BI et al. *JAMA Network Open.*
